# Evaluating the utility of syndromic case management for three sexually transmitted infections in women visiting hospitals in Delhi, India

**DOI:** 10.1038/s41598-017-01422-y

**Published:** 2017-05-03

**Authors:** Subash Chandra Sonkar, Kirti Wasnik, Anita Kumar, Vineeta Sharma, Pratima Mittal, Prashant Kumar Mishra, Mausumi Bharadwaj, Daman Saluja

**Affiliations:** 1 Medical Biotechnology Laboratory, Dr. B. R. Ambedkar Center for Biomedical Research, University of Delhi, Delhi, India; 2 Department of Obstetrics & Gynecology, Vardhman Mahavir Medical College and Safdarjung Hospital, New Delhi, India; 3 National Institute of Cancer Prevention and Research, Formerly Institute of Cytology and Preventive Oncology (Indian Council of Medical Research), Noida, Uttar Pradesh India

## Abstract

Utility of syndromic case management (SCM) in women visiting obstetrics & gynecology department needs to be evaluated as it is subjective and imperfect. Consequently, antibiotic resistance has accelerated along with increased risk of infection to the partners. To understand the effectiveness and/or inadequacies of SCM, 11000 women were recruited and examined by clinicians for infection by *Chlamydia trachomatis* (CT), *Neisseria gonorrhoeae* (NG), *Trichomonas vaginalis* (TV), *Bacterial vaginosis* (BV) and others. Amongst these patients, 1797 (16.3%) reported vaginal discharge (VD). Other symptoms included: vaginitis (97%), cervicitis (75%), genital ulcers (60%), abnormal vaginal discharge (55%) and lower abdominal pain (48%). The patients were treated for single or co-infections using pre-packed National Aids Control Program III STI/RTI Kits. However, based on PCR diagnostics, 1453/1797 (81%) subjects were uninfected for NG/TV/CT. Amongst 344 (19%) infected patients, 257 (75%) carried infection with single pathogen (TV/NG/CT) while 87/344 (25%) were co-infected with multiple pathogens. Prevalence of TV, NG & CT was 4%, 7% and 8% respectively. Co-infection with CT + NG was highest, 51% (44/87), whereas, co-infection with CT + TV was 21% and NG + TV was 18% while co-infection with all three pathogens was 1.3%. We conclude that SCM is imprecise and successful intervention requires accurate and confirmatory diagnostic approach.

## Introduction

Sexually transmitted diseases (STDs) caused by CT, NG and TV are common genitourinary infections and millions of new cases are reported annually in women of reproductive age worldwide^1, 2^. STDs continue to present major public health, social, and economic problem in developing countries leading to considerable morbidity, mortality and stigma^3^. Despite this, most infections in women often remain undiagnosed due to social stigma, asymptomatic nature of the infections and/or non-specific clinical presentations in patients. Amongst symptomatic cases, characterizations of the disease based on syndromic case management (SCM), and prediction that a patient is infected with a specific pathogen, relies on subjective judgment of the clinician^4–6^. The main presenting symptoms in majority of these patients is vaginal discharge along with others clinical symptoms. Therefore, accurate and early diagnosis of the infectious agent is of critical importance for timely intervention and to prevent further transmission of infection to their sexual partners. Another problem with common sexually transmitted infections (STIs) is the absence or poor symptoms in patients during early infection and hence they do not qualify for SCM^7–12^. The detection of infectious agents in such patients is difficult due to lack of screening strategies and poor performance (especially in terms of sensitivity) of the available rapid diagnostic tests. Hence most of the asymptomatic patients infected with STD are inadequately treated, or are never treated at all^11, 13, 14^. As a result, undiagnosed infections facilitate transmission of infection to their sexual partners as well as make the patient vulnerable to other STIs. Incorrect treatment due to similar symptoms by different microbes further complicates the disease management by syndromic approach as it can result in misdiagnosis. As a consequence, the patient is not treated for the existing infection, rather over-treated for some other pathogen. This has long-term consequences, as it may contribute towards antibiotic resistance^13, 15–22^. The inadequate public health programs coupled with ongoing socioeconomic and demographic trends have led to an epidemic of STIs in many developing countries. The development of antimicrobial resistance is a persistent problem and new antimicrobial agents are often much more expensive, thereby increasing the burden of disease management^23, 24^. The economic costs of follow up of these infections and their sequelae place a considerable burden on national health budgets and household income^25, 26^. Published reports from India by several authors show prevalence of various STI/RTIs in females but only very few studies had actually evaluated effectiveness of the syndromic approach in diagnosing STIs^7, 8, 10–14, 27, 28^. Many laboratory tests like real time PCR and Point of Care Test (POCTs) have been described recently but these are not often accessible to clinicians in practice due to their forbidding cost and/or due to the requirement of expertise^29–34^. Many patients although being symptomatic, fail to seek care, or delay in doing so, for personal reasons and/or social stigma. Thus, SCM is the best option for treating symptomatic patients during their first visit to clinic, it has inherent limitations as it relies on only symptomatic patients coming to clinical attention^35–38^. Diagnosis of STIs using molecular diagnostic tools and better POCT will pave way for faster treatment or provisions of intervention in infected patients and will thus prevent transmission to sexual partners or infants^39, 40^. Thus, there is an urgent need to evaluate the validity of SCM for enabling effective control efforts for CT, NG and TV, the three common STIs. The present study was undertaken with a specific objective of reporting detailed comparative analysis of effectiveness of SCM and PCR based diagnosis for CT, NG, and TV in disease management using 1797 clinical samples collected from multiple clinical sites.

## Materials and Methods

### Ethics statements

The study was carried out as per the institutional ethical guidelines and approval from; Dr. B.R. Ambedkar Center for Biomedical Research (ACBR) (ACBR No: F-50-2/Eth.com/ACBR/11/2107, Vardhman Mahavir Medical College (VMMC) and Safdarjung Hospital (No: 47-11-EC/30/51) and Institute of Cytology and Preventive Oncology (No. IEC/ICPO-ICMR/2011-P3). Informed written consent from all participants involved in the study was obtained. The results of the study did not influence the treatment. All patients were treated using National aids control organization- National aids control Program III (NACO-NACP III) STI/RTI Kits based on NACO guidelines (http://naco.gov.in/sti-rti-services).

### Recruitment of study subjects

Out of **11000** female patients who visited outpatient department (OPD) of obstetrics & gynecology of different hospitals, **1797** reported vaginal discharge and were recruited in the study as per the Institutional ethical guidelines and approval. Informed written consent was taken from each participating patient. **Inclusion criteria:** Patients recruited in the study were in the age group of 18 to ≥56 years with one or more of the following sign & symptoms (1) vaginal discharge, green to brown color frothy discharge, foul odor of discharge, (2) vaginal itching (3) edema or erythema (4) pruritus, genital ulcers (5) colpitis mascularis (strawberry cervix) by punctate hemorrhages (6) dysuria (7) pain during intercourse (8) urinary tract infection (9) soreness (10) vaginitis (11) lower abdominal pain (LAP) (12) elevated pH greater than 4.5 (normal pH of the vagina is 3.8–4.4) (13) presence of amines, vaginal leucocytosis, vulvar erythema, purulent with white blood cells (WBCs) (14) cervicitis (15) frequency of micturation, burning and pain on micturation. **Exclusion criteria:** Patients in the following category were not recruited: (1) age less than 18 years (2) Rh iso-immunization (3) use of antibiotics in the preceding two weeks (4) pregnancy (5) unmarried females. To confirm non-pregnancy every patient coming to Gynecology OPD was asked about her last menstrual period. If she was overdue, pregnancy test (urine based) was conducted in the OPD on the same day.

### Specimen collection and processing

A thorough clinical examination was done for lesions, warts, ectopic growth and vaginal/cervical discharge by the attending clinician. Cervical swab samples were collected and placed in the empty vial (dry swabs) and were kept frozen at −20 °C until use. Each cervical dry swab was incubated in phosphate buffered saline (PBS, 1 ml) for 10 minute at 4 °C, mixed by vortexing thoroughly to disperse the sample and then the cotton was squeezed. The sample (400 μl) was centrifuged at 11,000 × g at 4 °C for 10 minutes and the cell pellet was suspended in 40 μl of PBS followed by centrifugation at 11,000 × g at 4 °C for 5 minutes^13, 30, 33^. Total genomic DNA was isolated essentially as described earlier from our laboratory for other pathogens^13, 30, 33, 41–43^. The nucleic acids were stored at 4 °C till further use as template DNA for PCR assay and clinical evaluation.

### PCR amplification

All the patients were recommended antibiotic regime by clinicians in their OPD. Samples were also subjected to PCR for testing the presence of CT, NG and TV using specific PCR based assay as described in the published protocols^30, 41, 43^. A list of primer sets, specific to the genes of different microorganisms with corresponding amplicon size is given in Table 1. Precautions were taken to prevent PCR assay contamination by using separate rooms for clinical sample processing, PCR-setup and product detection. Furthermore, disposable plastic ware, aliquots of reagents, and autoclaved pipette tips were used. PCR amplification was carried out in 25 µl volume containing 1X Taq DNA polymerase buffer (50 mM KCl, 10 mM Tris-HCl pH 8.3, 1.5 mM MgCl_2_), 200 µM each of the four deoxynucleoside triphosphate (dNTPs) (Merck Specialties Private Ltd), 5 pmoles of forward and reverse primer each, 5 µl of total genomic DNA (gDNA) isolated from the clinical sample, 1.0 U of Taq DNA Polymerase (Bangalore Genei India Pvt. Ltd.). Each set of PCR assays included a negative control (sterile water instead of DNA template) and a positive control (1 ng purified gDNA isolated from CT, NG and TV culture). Amplification was performed in thermal cycler (I cycler, BioRad, USA) using different primer sets as per their reported protocols in the reference papers (see Table 1). The amplicons were analyzed by agarose gel electrophoresis (2%) in TAE buffer containing 0.5 μg/ml of ethidium bromide. After electrophoresis, the DNA bands were visualized on UV Transilluminator (Aplegen Inc, USA).

**Table 1:** List of primer sets specific for different pathogens used in the present study.

S. N.	Microorganisms	Gene	Primers Sequences	References
1	*C. trachomatis*	*gyrA*	C2 5′TGATGCTAGGGACGGATTAAAACC3′	41
			C5 5′TTCCCCTAAATTATGCGGTGGAA3′	
2	*N. gonorrhoeae*	*Orf1*	F 5′CAACTATTCCCGATTGCGA3′	43
			R 5′GTTATACAGCTTCGCCTGAA3′	
3	*T. vaginalis*	*pfoB*	F 5′CAAAGTCAACATGGCTATGAT3′	30
			R 5′GAAGACCTGTGTGGATGGATGT3′	

### Statistical analysis

The data was plotted and analyzed using the statistical software package SPSS version 20.

### Data analysis

A computational statistical test was used to analyze the distribution of symptomatic patients in reference to their clinical symptoms, age group and their infection/co-infection status.

## Results

### The syndromic versus laboratory diagnosis

**Figure: 1:**
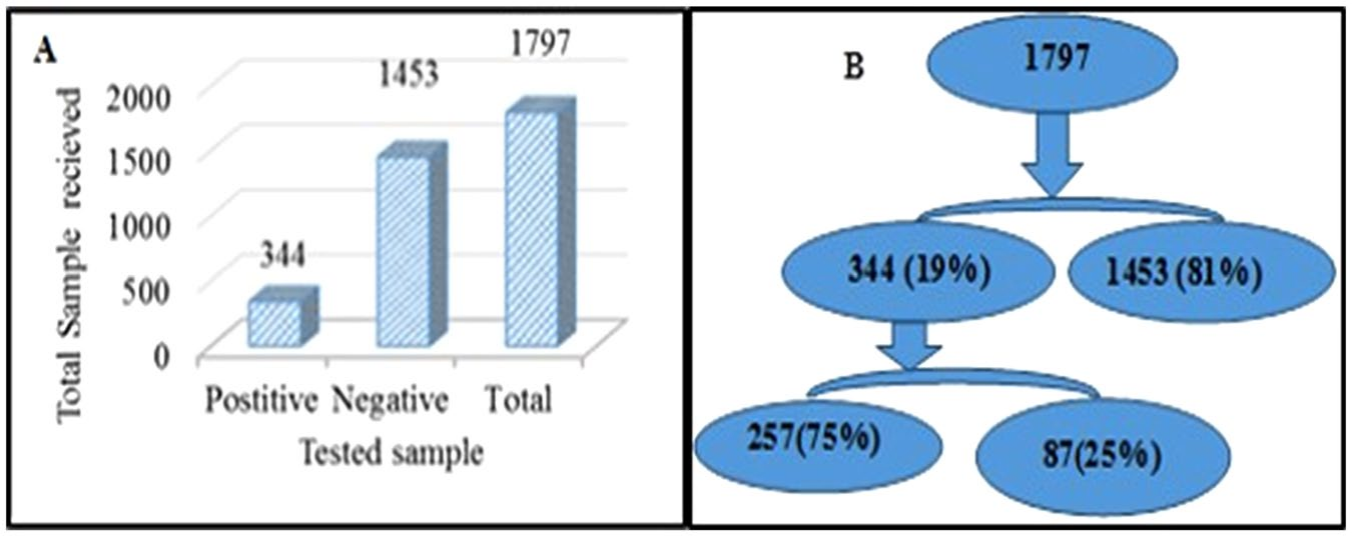
(**A**) Distribution of infected and uninfected patients based on PCR diagnostic assay using total genomic DNA isolated from clinical samples. (**B**) Diagrammatic representation of distribution of infected and uninfected patients for single or mixed infection. The distribution of infection with *T. vaginalis*, *N. gonorrhoeae* and *C. trachomatis* out of the total symptomatic patients enrolled in the study is shown.

During the period of **2011** to **2015** a total of 1797 new patients with complaint of VD and Pelvic Inflammatory disease (PID) attended OPD of Department of Obstetrics & Gynecology and were therefore recommended antibiotic treatment under SCM (Fig. 1A). All these subjects were also analyzed for CT, NG and TV infection using pathogen specific PCR amplification test as described under methods. Among the total 1797 samples, 344 (19%) subjects tested positive for one of the infections; CT/NG/TV whereas 1453 (81%) samples were negative for these three pathogens as determined by PCR based assay. Amongst the 344 infected patients, 257 (75%) women had single infection for CT, NG or TV while 87 (25%) subjects were co-infected with more than one pathogen; CT, NG or TV (Fig. 1B).

### Prevalence of *T. vaginalis*, *N. gonorrhoeae* and *C. trachomatis* infection

**Figure 2:**
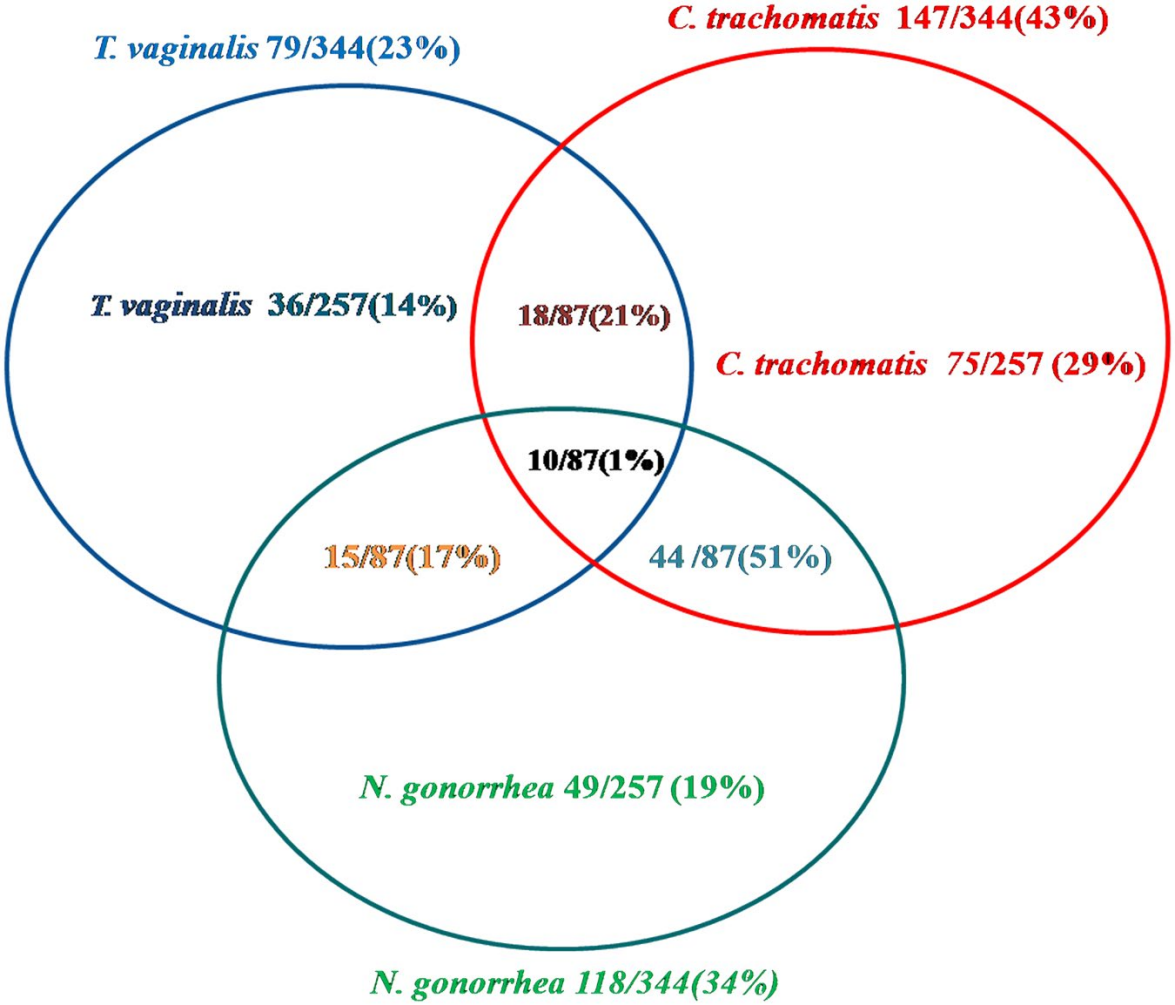
Venn diagram showing prevalence of *T. vaginalis*, *C. trachomatis* and *N. gonorrhoeae* infection as well as co-infection. DNA was isolated from samples collected from symptomatic patients and infection for these three pathogens was determined by PCR based diagnosis.

Based on the above results, we observed that the prevalence of TV, NG and CT infections in symptomatic women was 79/1797 (4%), 118/1797 (7%) and 147/1797 (8%) respectively. Out of the 344 infected samples, 87/344 (25%) patients had mixed infection with two or all the three pathogens tested. Among these, 44/87 subjects (51%) had co-infection of CT and NG, 18/87 subjects (21%) were co-infected with CT and TV while 15/87 subjects (18%) had NG and TV co-infection and 10/87 subjects (13%) were co-infected with all the three listed STIs (Fig. 2). It is also evident from these results that CT infection is more frequent compared to infection with NG^13^.

### Age specific prevalence and burden of *T. vaginalis*, *N. gonorrhoeae* and *C. trachomatis* infection

**Figure 3:**
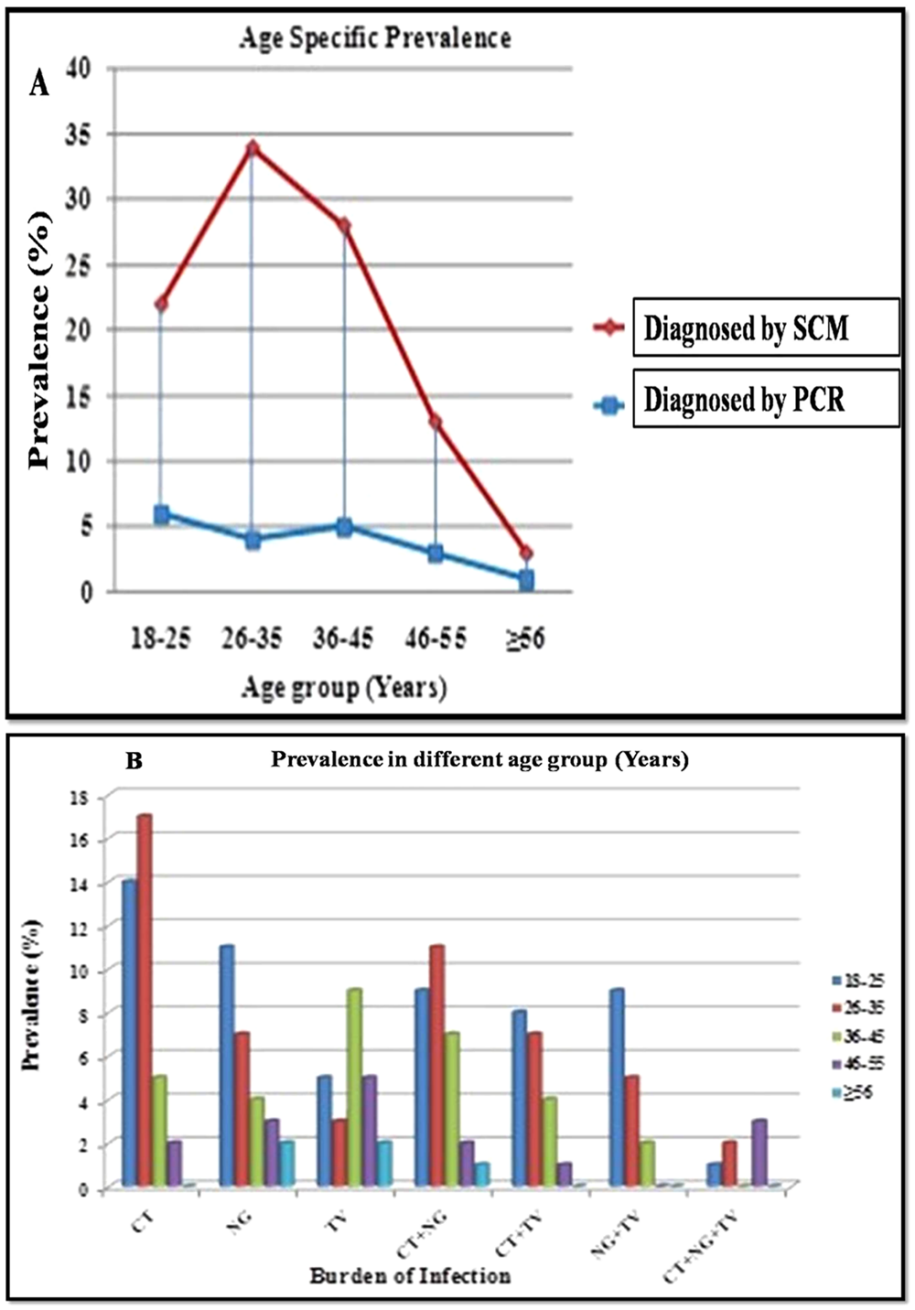
Age wise distribution of infection by *T. vaginalis*, *C. trachomatis* and *N. gonorrhoeae* as well as their co-infection as determined by PCR. (**A**) Percent prevalence was determined for patients infected with any of the three pathogens in different age groups as determined by PCR and SCM. (**B**) Bar diagram to show distribution of infection by *T. vaginalis*, *C. trachomatis* and *N. gonorrhoeae* alone as well as their co-infection in different age groups based on PCR diagnosis.

In this study, women above 18 years of age were further categorized into different age groups; 18–25, 26–35, (reproductive age groups), 36–45 (late reproductive age group), 46–55 (menopausal age group) & above 55. Figure 3A shows the distribution of infected patients who visited OPD, diagnosed based on SCM and PCR diagnosis. Based on the results of PCR, we observed that, the single infection either with CT alone or NG alone was more prevalent in reproductive age groups whereas infection with TV alone was observed more frequently in late reproductive group (Fig. 3B). Co-infection with CT & NG was also more common in subjects of reproductive age group as compared to patients in higher age groups (menopausal & above) where it is negligible. NG & TV co-infection was more prevalent in early reproductive age group. Triple infection with CT, NG & TV co-infection is low and nearly the same in all age groups except those above 55 years of age group (See Fig. 3B). Since the number of older women ≥56 years was limited, no conclusion could be drawn. However, we did observe that the infection with CT & NG was more prevalent among women in early reproductive age, but incidence of TV was higher in middle age group (36–55 years of age).

### Distribution of infection with *T. vaginalis*, *C. trachomatis* and *N. gonorrhoeae* infection based on syndromic approach

**Figure 4:**
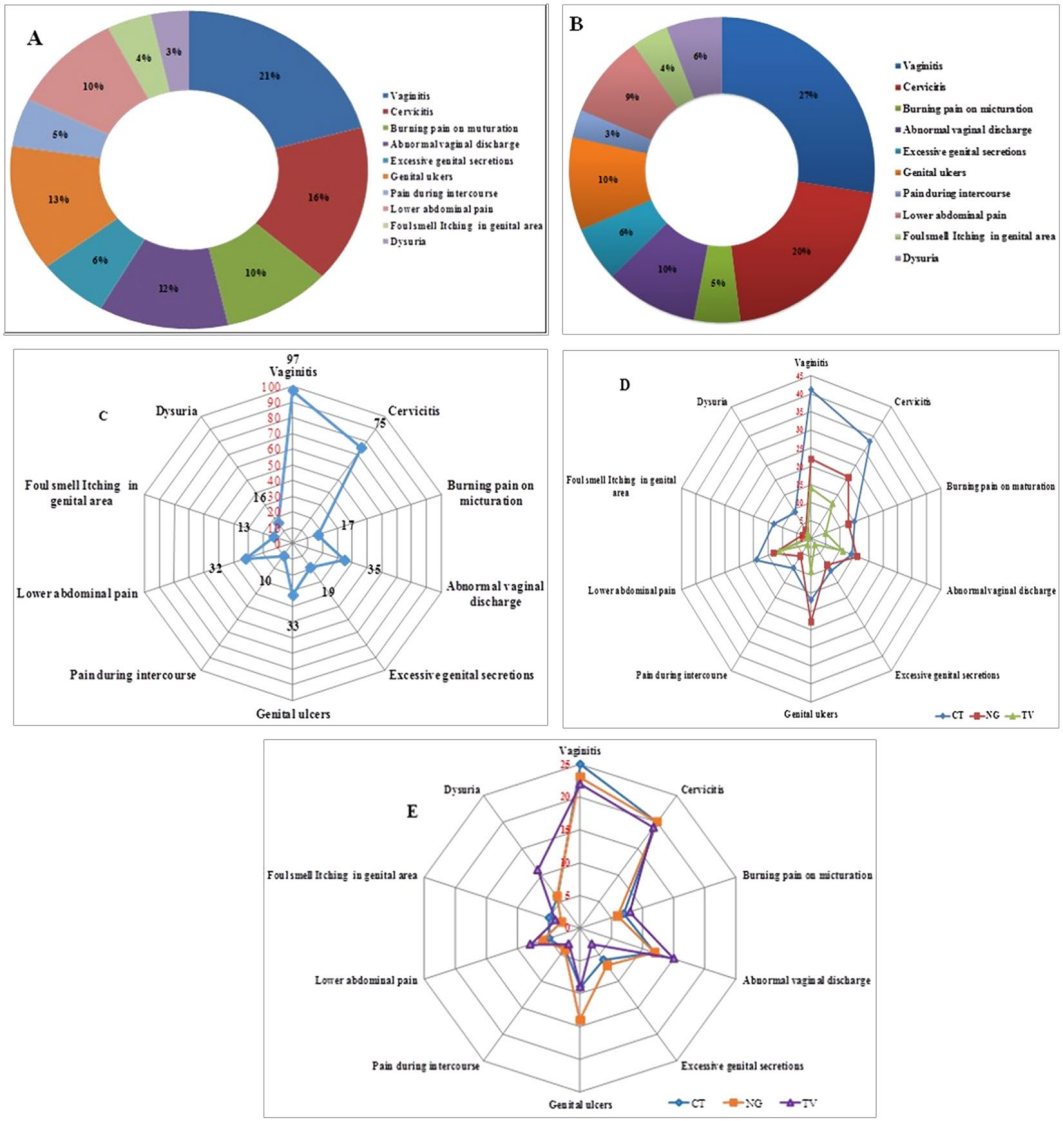
Percent distribution of different symptoms with reference to infections with *T. vaginalis*, *C. trachomatis* and *N. gonorrhoeae as determined by PCR diagnostics*. (**A**) Percentage of different symptoms amongst patients with vaginal discharge visiting the hospitals. (**B**) Percentage of different symptoms amongst patients tested positive for any of the three pathogens by PCR diagnostic assays. (**C**) Percent association of different symptoms among all the infected patients. (**D**) Relative association of different symptoms in patients infected with either *T. vaginalis*, or *C. trachomatis* or *N. gonorrhoeae* as determined by SCM. (**E**) Relative association of different symptoms in percent in patients infected with either *T. vaginalis*, or *C. trachomatis* or *N. gonorrhoeae* based on PCR diagnostics.

As per the guidelines of NACO for SCM, common symptoms are checked followed by specific clinical observations before prescribing the antibiotic treatment to the patients (see Table 2)^28, 29^. The most common symptoms as observed in these clinical samples, were vaginitis (21%), cervicitis (16%), genital ulcers (13%), abnormal vaginal discharge (12%), lower abdominal pain (10%), burning pain on micturation (10%), excessive genital secretion (6%), pain during intercourse (5%), foul smell, itching and dysuria etc. (see Fig. 4A). Since all these patients (n = 1797) were also subjected to PCR diagnostics, the distribution/association of these symptoms in patients infected with one or more pathogens, was also determined. As shown in Fig. 4B, we observed that the infection caused by any one of these three pathogens showed similar symptoms: vaginitis (97%), cervicitis (75%), genital ulcers (60%), abnormal vaginal discharge (55%), lower abdominal pain (48%) etc. (see Fig. 4C). In the distribution of clinical features with specific disease diagnosed by PCR and SCM, none of the symptoms were specifically associated with a given pathogen so as to provide us with discriminatory power for identification of a specific pathogen. In general, all of these pathogens present multiple symptoms upon infection (Fig. 4D). We also compared the relative percentage association of the different symptoms among patients infected with TV, CT or NG as determined by PCR diagnostics (Fig. 4E). Once again, we do not find any clear symptom that is specific to or associated with infection by TV, CT or NG.

**Table 2:** Management and treatment guidelines for symptomatic patients through syndromic case management.

S. No	STI/RTI Syndromic Diagonosis	KIT Prescribed	Name of the Drugs
1	Urethral Discharge/Ano Rectal Discharge/Cervical Discharge/Presumptive Treatment/Painful Scrotal Swelling	Kit 1 Gray	Azithromycin (1 g) OD STAT and Cefixime (400 mg) OD STAT
2	Vaginal Discharge (Vaginitis)	Kit 2 Green	Secnidazole (2 g) OD STAT and Fluconazole (150 mg) OD STAT
3	Genital Ulcer Disease‐ Non Herpetic	Kit 3 White	Benzathine penicillin (2.4 MU) IM STAT and Azithomycin (1 g) OD STAT
4	Genital Ulcer Disease‐ Non Herpetic(Allergic to Penicillin)	Kit 4 Blue	Doxycycline (100 mg) XBD X 14 DAYS and Azithromycin (1 g) X OD STAT
5	Genital Ulcer Disease‐ Herpetic	Kit 5 Red	Acyclovir (400 mg)X TDS X 7 DAYS
6	Lower Abdominal Pain (Pelvic Inflammatory Disease)	Kit 6 Yellow	Cefixime (400 mg) X OD STAT and Metronidazole (400 mg) X BD X 14 DAYS and Doxycycline (100 mg) X BD X 14 DAYS.
7	Inguinal Bubo	Kit 7 Black	Doxycycline (100 mg)X BD X 21 DAYS and Azithromycin (1 g) X OD STAT

## Discussion

STI continues to present a major public health, social, and economic problem in developing countries, leading to considerable morbidity, mortality and social stigma. Millions of new cases of STDs due to TV, CT and NG are reported annually worldwide and are the major cause of genitourinary infections especially amongst women in reproductive age group^44–53^. Earlier studies have also shown that sexually transmitted infections increase the chance of HIV infection by 2–5 folds^54–56^. Due to lack of epidemiological studies, the true numbers of infection and prevalence in developing countries is not available. This is further exacerbated due to the fact that most STI in women frequently remain unrecognized due to asymptomatic nature of infection and obscure clinical presentations in the patient. Characterization and/or screening of the diseases caused by these pathogens, based on the signs and symptoms using SCM guidelines proposed by WHO-NACO-NACP-III, is neither accurate nor confirmative. However, it allows an experienced clinician to recommend treatment to the patients in their first visit and hence increases the chance of cure and reduction in onward transmission to the sexual partner. Nevertheless, clinical spectrum of infection is broad and several STIs generate similar clinical spectra. So the symptom based treatment for the infecting microbe is challenging with the risk of misdiagnosis. This lack of accurate and specific diagnosis leads to predefined antimicrobial prescriptions which is not only the cause of over treatment, but contributes to acquiring antibiotic resistance. The main presenting symptom in majority of the patients infected with TV, CT and NG is vaginal discharge which when remain untreated may result in PID, infertility, ectopic pregnancy and many other complications. Therefore, correct and early diagnosis of these diseases is of critical importance for the timely medical intervention. Since the management of these diseases in low resource settings is based on subjective judgment, a large pool of patients, which is asymptomatic, remains untreated and serves as a reservoir for further spread of the disease. Hence feasible screening strategies to identify infection amongst asymptomatic patients in developing countries are also urgently required.

Despite the availability of several diagnostic assays, ranging from microscopy to real time PCR, resource limited countries including India have relied on SCM. This has happened due to high cost of the available diagnostic kits and lack of sophisticated infra-structure. Use of a simple, inexpensive diagnostic assay can prevent overtreatment and waste of scarce resources to a large extent. As a step in this direction, the present study evaluates the diagnostic accuracy of the PCR based assay against SCM used in diagnosis of VD in symptomatic women. STIs are among the top five reasons for which adults seek medical care. Hence, to cope with the disease, SCM is still preferred in India in-spite of its short comings as it is the cheapest way to seek and impart treatment. To check the utility of SCM we compared the actual number of patients who were genuinely infected, as evident by PCR based diagnostics, versus patients diagnosed for TV, CT and NG using SCM. Our results clearly establish that the SCM is imprecise, inaccurate and results in high percentage of overtreatment of patients. This may contribute to developing resistance to common antibiotics. In order to find out which reproductive age group is most vulnerable to infection based on symptoms, the samples were segregated in different age groups (18 to more than 55 years). Among all the age groups, the distribution of complaints was similar; suggesting that VD and other symptoms are not reliable features for predicting the cause of infection^57–60^. However, based on their complaints, physical examination, and diagnosis 26–35 years age group and 36–45 years age group were found to be most prone to infection. This is understandable since 26 to 45 years of age represents the active reproductive phase amongst women as reported earlier^13^. Our studies thus suggest that SCM is unsuitable for routine clinical diagnosis and management of TV, CT and NG. One of the reasons for this is that the spectrum of infection is broad and other STIs have similar clinical presentations and hence it is difficult to differentiate and characterize the infectious agent by the routine clinical examination.

NACO developed a simplified tool (a flowchart or algorithm) to guide health workers in the implementation of SCM of STI for women with symptoms of VD and/or LAP. However, it is important to recognize the limitations of the VD algorithms, particularly in the management of cervical (gonococcal and chlamydial) infections. In general, but especially in low prevalence settings and in adolescent females, endogenous vaginitis rather than STI is the main cause of VD. While attempts have been made to increase the sensitivity and specificity of the vaginal discharge algorithm for the diagnosis of cervical infection, through the introduction of an appropriate, situation-specific risk assessment, both remain low. Moreover, some of the risk assessment questions based on demographics, religion, income, illiteracy, occupations, marital status and pregnancy tend to incorrectly classify too many adolescents at risk of cervical infection^61–63^. Symptoms-directed characterization of infectious agent that is TV, CT and NG is difficult as the symptoms are similar e.g., vaginitis, cervicitis, burning pain on micturation, abnormal vaginal discharge, excessive genital secretions, genital ulcers, pain during intercourse, foul smell, itching in genital area and dysuria in women. The control of these infections can lead to substantial reduction in the transmission of other STIs as well. Thus, while in resource poor settings, SCM is the only option for treating symptomatic patients and is a good tool for STD control, it has inherent limitations. Based on the present study, we therefore propose that improvements in guidelines for SCM are needed for confirmative and accurate diagnosis to minimize misdiagnosis and overtreatment. Alternatively, it is important to switch to more accurate diagnostic methods such as PCR, LAMP or other POCT. Moreover, there is need to develop low cost, easy to use nucleic acid based diagnostic methods that can be used in poor resource settings by untrained workers. This shall not only prevent transmission of infection to their sexual partner or infants but help in reducing the rampant use of antibiotics.

## Conclusion

Our results clearly demonstrate that the prevalence of CT and NG is still significant among female patients visiting Obstetrics & Gynecology Departments. The study underpins the need to conduct diagnostic assays for identification of causative pathogen before implementing antibiotic treatment to patients with vaginal discharge. It also divulges the need to review the use of syndromic case management for controlling sexually transmitted diseases in India.
